# Genetic Polymorphism in Wine Yeasts: Mechanisms and Methods for Its Detection

**DOI:** 10.3389/fmicb.2017.00806

**Published:** 2017-05-04

**Authors:** José M. Guillamón, Eladio Barrio

**Affiliations:** ^1^Departamento de Biotecnología de los Alimentos, Instituto de Agroquímica y Tecnología de Alimentos – Consejo Superior de Investigaciones Científicas (CSIC)Valencia, Spain; ^2^Departamento de Genética, Universidad de ValenciaValencia, Spain

**Keywords:** SNP, insertions, deletions, ploidy changes, interspecific hybridization, gene horizontal transfer, PCR-based methods, NGS

## Abstract

The processes of yeast selection for using as wine fermentation starters have revealed a great phenotypic diversity both at interspecific and intraspecific level, which is explained by a corresponding genetic variation among different yeast isolates. Thus, the mechanisms involved in promoting these genetic changes are the main engine generating yeast biodiversity. Currently, an important task to understand biodiversity, population structure and evolutionary history of wine yeasts is the study of the molecular mechanisms involved in yeast adaptation to wine fermentation, and on remodeling the genomic features of wine yeast, unconsciously selected since the advent of winemaking. Moreover, the availability of rapid and simple molecular techniques that show genetic polymorphisms at species and strain levels have enabled the study of yeast diversity during wine fermentation. This review will summarize the mechanisms involved in generating genetic polymorphisms in yeasts, the molecular methods used to unveil genetic variation, and the utility of these polymorphisms to differentiate strains, populations, and species in order to infer the evolutionary history and the adaptive evolution of wine yeasts, and to identify their influence on their biotechnological and sensorial properties.

## Introduction

During the advent of agriculture, humans learnt to put to good use spoiled fruit juices that spontaneously fermented in order to produce alcoholic beverages (Mortimer, 2000), of which grape wine is one of the oldest (McGovern et al., 1997). Alcoholic fermentation of grape must to wine is a complex process that involves the sequential development of microorganisms, mainly yeasts, but also filamentous fungi, lactic acid bacteria and acetic acid bacteria (Pretorius, 2000). Several dozens of yeast species may be present in early wine fermentation stages. However, the yeast population progressively becomes dominated by yeasts that belong to the *Saccharomyces* genus, mainly *Saccharomyces cerevisiae* as alcohol concentration increases (Fleet and Heard, 1993).

Yeasts from the *Saccharomyces* genus exhibit distinctive physiological properties that are not found in other yeasts (Vaughan-Martini and Martini, 2011). The most important is their excellent ability to ferment sugars vigorously to produce alcohol under both aerobic and anaerobic conditions (Piškur et al., 2006; Dashko et al., 2014). This aptitude allows them to quickly colonize sugar-rich substrates and outcompete other yeasts that are much less tolerant to the ethanol and heat produced during fermentation (Goddard, 2008; Salvadó et al., 2011). Consequently, wine *S. cerevisiae* strains are highly specialized organisms that have evolved to utilize the different environments or ecological niches provided by human activity. This process can be described as “unaware domestication” and is responsible for the peculiar genetic characteristics of these yeasts (Fay and Benavides, 2005; Liti et al., 2009; Almeida et al., 2015). *S. cerevisiae* strains that exhibit high ethanol tolerance and excellent fermentative ability are extensively used in winemaking as starter cultures. However, a side-effect of the widespread use of these commercial starter cultures is the elimination of native microbiota, which might result in wines with similar analytical and sensory properties, depriving them from the variability and diversity that define the typicality of a wine. Nonetheless, a way of balancing control and yeast population diversity during wine fermentation is the selection of non-*Saccharomyces* yeasts with optimal oenological traits.

Thus, in recent years, other wine yeast species attracted much interest for their potential application to solve new challenges in the winemaking industry generated by consumer demands of aromatic wines with lower ethanol contents, or due to the modification of the composition and properties of grape must because of climate change (Jones et al., 2005). New yeast starters from other *Saccharomyces* species, and from non-*Saccharomyces* species, are being developed to be used in mixed cultures or in sequential inoculations in order to direct fermentations to obtain wines with higher glycerol concentration and aroma intensity, and lower ethanol and acetic acid, contents. In this way, alternative *Saccharomyces* species, such as *S. uvarum* and *S. kudriavzevii*, and their hybrids with *S. cerevisiae*, exhibit good fermentative capabilities at low temperature, and produce wines with lower alcohol concentration, higher glycerol amounts, and excellent aromatic profiles (González et al., 2007; Gamero et al., 2013; Peris et al., 2016), properties of great interest for the wine industry. Additionally the use of non-*Saccharomyces* species, such as *Metschnikowia pulcherrima*, in co-cultures with *S. cerevisiae* has been suggested as an enological practice to reduce ethanol contents in wine (Contreras et al., 2014; Morales et al., 2015). The use of *Candida zemplinina, Hanseniaspora vineae*, and *Torulaspora delbrueckii* yeasts has been proposed to improve the organoleptic properties of wines (Renault et al., 2009; Medina et al., 2013; Jolly et al., 2014).

The study of natural yeast isolates, both at interspecific and intraspecific level, has revealed a great phenotypic diversity, which is explained by a corresponding genetic variation. Thus, the mechanisms involved in promoting these genetic changes are the main engine driving yeast biodiversity. Currently, an important task to understand biodiversity, population structure and evolutionary history of wine yeasts is the study of the molecular mechanisms involved in yeast adaptation to the industrial process, and on remodeling the genomic features of wine yeast, unconsciously selected since the advent of winemaking (Barrio et al., 2006; Marsit and Dequin, 2015). Genetic variation is the ultimate source of heritable variation, acted upon by evolutionary forces such as selection and genetic drift. The neo-Darwinian theory of evolution by natural selection was founded on the notion that natural populations hold abundant genetic polymorphisms to respond to selection. This genetic variability is due to the occurrence of different alleles originated by mutation and homologous recombination. Adaptation is then the result of the gradual accumulation of minor changes in allele frequencies due to the action of natural selection. Different molecular approaches have shown that mutations include not only the generation of new alleles by nucleotide changes, but also the acquisition of new genes or the formation of radically different alleles originated by other mechanisms.

This article reviews the mechanisms involved in generating genetic polymorphisms in yeasts, the molecular methods used to unveil genetic variation, and the utility of these polymorphisms to differentiate strains, populations, and species in order to infer the evolutionary history and the adaptive evolution of wine yeasts, and to identify their influence on their biotechnological and sensorial properties.

## Mechanisms Involved in the Generation of Yeast Genetic Polymorphisms

**FIGURE 1:**
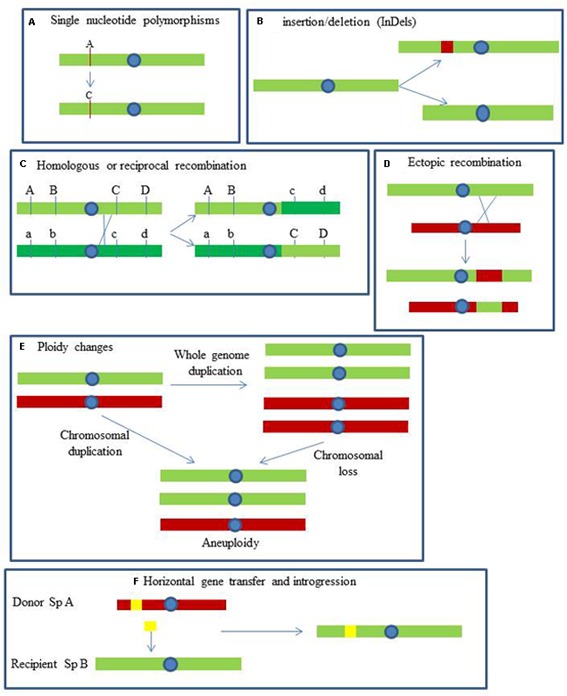
**Mechanisms involved in the generation of yeast genetic polymorphism.** **(A)** Single nucleotide polymorphisms: changes in single nucleotides. **(B)** InDels: insertion and deletion events of relatively short pieces of DNA. **(C)** Homologous or reciprocal recombination: gene conversion by crossing-over between homologous chromosomes. **(D)** Ectopic recombination: unequal crossing-over between non-homologous loci. **(E)** Ploidy changes: the whole genome, or large parts, is duplicated or lost. **(F)** Horizontal gene transfer: transfer of genes by alternative means to sexual reproduction (adapted from Steensels et al., 2014).

Yeast genomes are exposed to dynamic mechanisms generating genetic polymorphisms with different evolutionary consequences. These mechanisms can be classified in single nucleotide polymorphisms (SNPs), short sequence insertions or deletions, recombination and gene conversion, short tandem duplications, gene and segmental duplications, gross chromosomal rearrangements (GCRs), ploidy changes and interspecific hybridization (**Figure 1**).

### Single Nucleotide Polymorphisms

Single nucleotide polymorphisms are single nucleotide positions in DNA at which different sequence alternatives (alleles) exist in individuals of the same population or species (Brookes, 1999). More broadly speaking, they correspond to the single nucleotide substitutions or small nucleotide insertion–deletions (indels) generated by point mutation due to errors in DNA replication or DNA repair. Although SNPs are less variable than microsatellites (see below), they represent the most widespread type of sequence variation in genomes. SNPs are presently inferred mainly from single gene, multilocus, and genome sequence comparisons (Ben Ari et al., 2005; Fay and Benavides, 2005; Liti et al., 2009; Hyma and Fay, 2013).

Nucleotide polymorphisms have also emerged as valuable genetic markers to reveal the evolutionary history of populations. In this way, SNPs from genome sequence analyses have been used to determine phylogenetic relationships among *S. cerevisiae* strains (Liti et al., 2009; Almeida et al., 2015; Gallone et al., 2016) and other *Saccharomyces* species (Almeida et al., 2014; Peris et al., 2014).

Nucleotide polymorphisms in coding regions or regulatory sequences may change protein structure and function or modify gene expression. Therefore, sequence analyses can also be useful to unveil adaptive evolution in yeasts. In their study, Aa et al. (2006) also reported the presence of a greater replacement polymorphism in gene *SSU1*, which provided evidence for diversifying selection by acting on its protein product, a sulfite exporter involved in sulfite resistance, as a response to the selective pressure imposed by employing sulfite in winemaking as a bactericide.

Nucleotide divergences in promoter regions may have major effects on gene expression levels, which can also be affected by nucleotide changes in the coding regions of transcription factor genes. In a comparative genome analysis searching for promoters with divergent function, Engle and Fay (2012) identified changes in both the coding and upstream non-coding sequences of yeast transcription factor gene *FZF1*, which resulted in differences to confer sulfite resistance. Non-coding changes affected transcription factor expression, whereas coding changes affected the expression of *SSU1*, the sulfite pump.

In some cases, polymorphisms have been demonstrated as being involved in generating phenotypic variation in yeast properties of biotechnological interest. By way of example, Marullo et al. (2007) studied the genetic basis for variations in acetic acid production in wine strains by quantitative trait loci (QTL) mapping. They showed that this variation was due to a non-synonymous single-nucleotide polymorphism in *ASP1*. The corresponding amino acid replacement abolished the catalytic activity of encoded asparaginase type I, which affected nitrogen assimilation, the CO_2_ production rate and acetic acid production. Guillaume et al. (2007) also described nucleotide substitutions in gene *HXT3*, which encodes one of the hexose transporters, that resulted in improved fructose assimilation during wine fermentation. Oliveira et al. (2014) observed that non-synonymous nucleotide divergences between *GPD1* genes from *S. kudriavzevii* and *S. cerevisiae* could explain differences in the *V*_max_ of glycerol-3-phosphate dehydrogenases, responsible for higher glycerol production in *S. kudriavzevii* (Arroyo-López et al., 2010).

In some extraordinary cases, missense and nonsense mutations can take an adaptive value. Will et al. (2010) showed that independent loss in *S. cerevisiae* strains of paralogous *AQY1* and *AQY2* genes, which encode the water-transporter aquaporins involved in freeze-thaw tolerance, provided a major fitness advantage in highly sugar-rich environments.

### Microsatellites

Microsatellites, simple sequence repeats (SSR) or short tandem repeats (STR) are direct duplications of short motifs of nucleotides arranged in tandem, which display variation in the number of repeats. The high polymorphism of microsatellites is due to the relatively high motif insertion/deletion (InDels) rates generated by slipped-strand mispairing between contiguous repeats during replication, and by unequal crossover between motifs.

The sequence that surrounds the repeat region is usually conserved, and allows the design of PCR primers to amplify the repeat region. Differences in the number of repeats are identified as length polymorphisms in PCR products by using high-resolution electrophoresis, including automatic DNA sequencers. Microsatellite codominant polymorphisms have proven very useful for strain discrimination (González-Techera et al., 2001; Pérez et al., 2001; Legras et al., 2005; Masneuf-Pomarède et al., 2007), for the genetic analysis of yeast populations (Ayoub et al., 2006; Legras et al., 2007), and to also determine levels of heterozygosity and ploidy (Bradbury et al., 2006; Katz Ezov et al., 2006).

Microsatellites variation may affect phenotypic traits only when located in regulatory and coding regions. With yeasts, the most important source of gene elongation is the presence of codon repeats generated by trinucleotide expansions. No correlation between these expansions and variation in yeast traits of industrial interest has yet been demonstrated. However, a higher frequency of codon repeats in transcription factors and protein kinases has been described in yeasts (Richard and Dujon, 1997; Albà et al., 1999). Changes in the length of repeats in such cellular components of the cell signaling system could alter their biochemical properties, and therefore readjust their interactions with regulatory DNA regions or with other transcription factors, which could provide evolutionary divergence (Albà et al., 1999; Malpertuy et al., 2003).

### Meiotic, Mitotic Recombination, and Levels of Heterozygosity

Homologous or reciprocal recombination and gene conversion due to equal crossing-over between homologous chromosomes are the main mechanisms that generate new combinations of mutations. A non-reciprocal recombination due to unequal crossing-over is the source of the duplications, deletions, and translocations that may be involved in the generation of novelties, as reported in the following sections.

In diploid *Saccharomyces* yeasts, the frequency and nature of recombination during sexual, and also asexual, reproduction have an important impact on their patterns of variability. Recombination occurs during both meiosis and mitosis, although meiotic recombination is about 1000 times more frequent. The analysis of recombination rates and linkage disequilibrium using molecular markers provides interesting information about sexual reproduction frequency in yeasts (Koufopanou et al., 2006; Kuehne et al., 2007; Magwene et al., 2011; Gallone et al., 2016).

Mortimer (2000) observed that natural populations *S. cerevisiae* from wine fermentations and vineyards were diploid, homothallic and showed a low genetic diversity correlated with their high fertility. These observations led the authors to propose a mechanism of evolution for these wine yeasts, named as “genome renewal”. This mechanism is based on the ability of homothallic haploid *S. cerevisiae* cells to switch their mating type during mitosis, followed by a mother–daughter mating. This way, strains of *S. cerevisiae*, accumulating heterozygous recessive mutations during long periods of asexual reproduction, can change to completely homozygous diploids, except for the *MAT* locus, after a single sexual cycle followed by a homothallic switching of the haploid spores. This process, called haploselfing or autodiploidization, promotes the action of selection, by removing recessive deleterious genes and fixing recessive beneficial alleles, thereby enabling yeasts to adapt efficiently to changing environmental conditions. However, mitotic recombination or gene conversion during vegetative growth (Puig et al., 2000) as well as break-induced replication (Pâques and Haber, 1999) also promote loss of heterozygosity (LOH) in diploid wine *S. cerevisiae* cells (Ramírez et al., 2004). The direction of the LOH is asymmetrical in heterozygous yeasts due to the mechanisms involved, but the speed of the process increases as a consequence of the higher viability of the new homozygous yeasts with respect to the original heterozygous cells, which promotes a rapid asymmetric evolution in wine yeasts (Ambrona et al., 2005).

Ruderfer et al. (2006) developed a method to estimate the outcrossing rate in *S. cerevisiae* from whole-genome sequences from three strains and one of their sibling species, *S. paradoxus*. Based on recombination patterns, they estimated that the outcrossing rate was very low in yeasts as it occurred only once every 50000 divisions, which suggested that sex in yeast primarily involves inbreeding via intratetrad mating or haploselfing.

Many population genomic studies (Liti et al., 2009; Almeida et al., 2015; Strope et al., 2015) were based on homozygous strains derived from monosporic cultures, which make impossible to characterize the genome heterozygosity. Nonetheless, the presence of clinical and industrial mosaic strains suggested a significant admixture between *S. cerevisiae* lineages.

Sequencing of new clinical, environmental, and industrial isolates of *S. cerevisiae* unveiled a high number of heterozygous positions across the genomes of clinical and industrial yeasts (Argueso et al., 2009; Akao et al., 2011; Borneman et al., 2011; Magwene et al., 2011; Gallone et al., 2016) in contrast to *S. cerevisiae* isolated from wild environments such as oak forests from North America and Asia, which show very low levels of heterozygosity (Kuehne et al., 2007; Wang et al., 2012). Magwene et al. (2011) proposed that the high levels of heterozygosity observed in clinical and industrial strains most likely resulted from outcrossing between genetically diverse lineages, mediated by unaware strain trafficking due human activity. In addition to the presence of mosaic monosporic strains (Liti et al., 2009), this is also supported by the observation of two populations of *S. cerevisiae*, native and introduced wine strains, coexisting and interbreeding in Cachaça fermentations (Badotti et al., 2014).

Yeast outcrossing likely occurs in natural environments because sexual reproduction has not been observed in fermentation environments (Puig et al., 2000), and several authors (Pulvirenti et al., 2002; Stefanini et al., 2016b) showed that the insect gut provides the appropriate conditions for sporulation, germination, and mating of *Saccharomyces* strains.

Magwene et al. (2011) also proposed that these high levels of heterozygosity coupled with clonal expansion and selfing during rare sexual cycles generate a very large number of new homozygous allelic combinations facilitating rapid adaptation to the novel environments created by human activity. The lower levels of heterozygosity in wine yeasts compared to other industrial yeasts, such as brewing yeasts, suggest that these rare sexual cycles, favored by nutrient depletion, seem to be more frequent in wine yeasts (Borneman et al., 2016; Gallone et al., 2016). However, Ambrona and Ramírez (2007) observed after sporulation of wine yeasts that the frequency of mating between cells from the same ascus, favored by physical proximity, was higher than haploselfing and than mating between germinated haploid cells from different tetrads. This mating restriction slowed down the LOH process of the wine yeast population, maintaining the heterozygosity lower than would be expected by outcrossing but higher than expected under the Mortimer genome renewal model.

### Gene and Segmental Duplications

Gene duplication is the most important source of new genes in eukaryotes. Paralogs are redundant gene copies generated by duplication. Paralogs are unrestricted to preserve their original function and, therefore, can undergo divergent evolution resulting in novel gene functions.

Gene duplications can be produced by different mechanisms to result in the duplication of a single gene or group of adjacent genes (Koszul et al., 2006) in chromosome duplication, called aneuploidy (Hughes et al., 2000), or in the duplication of the whole genome content, called polyploidy (Wolfe and Shields, 1997).

In some cases, redundant genes can be retained if there is an evolutionary advantage to having extra dose repetitions. In others, one duplicate is free to accumulate mutations because only one of the duplicates is under purifying selection due to constraints to preserve the ancestral function. The classical Dobzhansky–Muller model, of generation of novel genes by duplication, postulates that a pair of paralogs is preserved if one of the copies gains a new function while the other maintains the original role. Nevertheless, this process, called neo-functionalization, is expected to be particularly unusual because beneficial mutations resulting in a new function are very rare comparing to loss-of-function mutations, which can be neutrally fixed in the unrestricted copy. As a result, the redundant duplicate finally becomes a non-functional gene, a process known as non-functionalization. According to the classical model, the presence of paralogous genes in the genome would be rare in the long term, however, the sequencing of complete yeast genomes showed that the preservation of duplicates is quite frequent (Wagner, 1998).

Force et al. (1999) suggested an alternative mechanism to explain the retention of paralogous genes. This process, called sub-functionalization, requires an ancestral gene with more than one function, which are independent lost in the paralogous genes by complementary degenerative mutations. This model requires that both duplicates complement their preserved subfunctions to produce the full patterns of activity of the ancestral gene. Subsequently, the adaptive evolution can promote the subfunctional specialization of each paralogous gene.

One of the best known examples of subfunctionalization in yeasts is the *GAL1-GAL3* paralogous pair, present in *Saccharomyces* species (Hittinger and Carroll, 2007). The *GAL1* gene codes for a galactokinase that catalyzes the phosphorylation of α-D-galactose to α-D-galactose-1-phosphate in the first step of galactose catabolism, while the galactose-inducible *GAL3* gene encodes a transcriptional regulator involved in activation of the *GAL* genes, including *GAL1*, in response to galactose. *Kluyveromyces lactis* possesses one single *GAL1* gene coding for a protein with both functions, transcriptional regulator and galactokinase. The phylogenetic analysis of their sequences indicates that *Saccharomyces GAL1–GAL3* genes duplicated after the divergence of *K. lactis GAL1*, and subsequently, each paralogous gene specialized by subfunctionalization in one of the original functions.

The most frequent events of gene duplications are those that involve a single gene or group of adjacent genes, called segmental duplication. Different mechanisms have been postulated to explain the origin of single-gene and segmental tandem duplications. The critical step lies in the origin of first tandem duplication, which requires the presence of similar nucleotide sequences to flank the duplicated region. These similar sequences may also be provided by transposable elements. An ectopic recombination between homologous chromosomes, or an unequal sister chromatide exchange at similar sequences, also results in genome region duplications. Subsequent duplications can occur by unequal non-homologous recombination between paralogous repeats (Zhao et al., 2014), which gives rise to tandemly repeated multigene families.

Yeast genomes encode hundreds of multigene families with three or more duplicated genes, which indicate that successive single gene or segmental duplications should have occurred. A comparative genome analysis (Dujon et al., 2004) reveled that tandem gene duplications are very frequent, and have occurred during the evolution of hemiascomycetous yeasts.

Different examples of segmental duplications are dispersed throughout the genome. One of them is the *CUP*1 tandem cluster, located on chromosome VIII, that encodes a copper metallothionein involved in cupper resistance (Welch et al., 1983). Gene copy number variations were generated by unequal non-homologous recombination (Zhao et al., 2014), and are clearly associated with cupper resistance differences (Warringer et al., 2011).

Other gene families are in the subtelomeric regions located nearby chromosome telomeres. Most subtelomeric gene families encode proteins involved in cell membrane and cell wall components, such as lectin-like proteins (*FLO* genes), sugar transporters (*HXT*), genes related to cell–cell fusion (*PRM*), and the assimilation and utilization of nutrients (*GAL*, *MAL*, *SUC*, and *PHO*), etc. (Carlson and Botstein, 1983; Ness and Aigle, 1995; Liti and Louis, 2005; Voordeckers et al., 2012).

Although these genes are not essential, they can be important for yeast adaptation to new environmental conditions. This way, genomic churning due to an ectopic recombination between repeated subtelomeric regions plays a key role in rapidly creating phenotypic diversity over evolutionary time, which favors the rapid adaptation of yeasts to industrial environments (Brown et al., 2010; Christiaens et al., 2012; Voordeckers et al., 2012).

### Chromosomal Rearrangements

The analysis of chromosomal DNA by pulse field gel electrophoresis (PFGE) has revealed important chromosome length polymorphisms in yeasts (Bidenne et al., 1992; Querol et al., 1992; Schütz and Gafner, 1994; Nadal et al., 1999). These polymorphisms are due to GCRs, such as translocations, inversions, duplications, and deletions of large chromosomal regions.

The comparative analysis of chromosomes and genomes (Fischer et al., 2000; Infante et al., 2003; Kellis et al., 2003; Dunn et al., 2005) has shown that duplicated genes, transposable elements and dispersed tRNA-encoding genes are found at chromosomal breakpoints, which supports unequal non-homologous recombination as the mechanism implicated in the origin of GCR. Actually, Ty elements or δ-LTRs are well known as favoring genome instability by ectopic recombination in yeasts (Rachidi et al., 1999; Infante et al., 2003). Unequal non-homologous recombination between sequences of high similarity present in non-homologous genes, between duplicated genes, or between Ty retrotransposons could generate evolutionary novelties, such as new chimerical genes with a modified function or with changes in their regulation (Christiaens et al., 2012; Marsit et al., 2015).

Industrial yeasts exhibit GCR associated to differences in physiological properties of industrial importance (Codón and Benítez, 1995), which is indicative of their potential role in the adaptation of yeasts to industrial environments. As examples, the fact that the same translocation in a region adjacent to *CIT1*, involved in tricarboxylic acid cycle regulation, repeats in different strains that have evolved under growth in glucose-limited chemostats is indicative of its adaptive value (Dunham et al., 2002). Competition experiments between *S. cerevisiae* strains with artificial translocations under different physiological conditions (Colson et al., 2004) have shown that translocated strains consistently outcompete the reference strain with no translocation.

**FIGURE 2:**
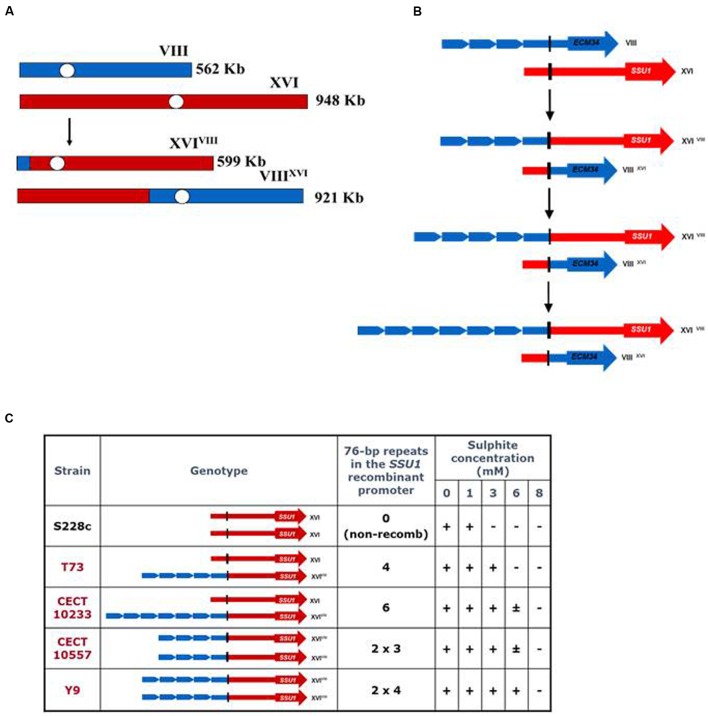
**Mechanism involved in wine yeast tolerance to sulfite: the gene *SSU1*, a paradigm of adaptive genome rearrangement.** **(A)** Reciprocal translocation between chromosomes VIII and XVI observed in wine yeast strains. **(B)** Organization of the *ECM34* and *SSU1* non-recombinant alleles and their corresponding recombinant variants obtained by an illegitimate crossing-over. **(C)** Sulfite tolerance of yeast strains exhibiting different numbers of repeats of a 76-bp region in the recombinant *SSU1* promoter (adapted from Pérez-Ortín et al., 2002).

Pérez-Ortín et al. (2002) demonstrated that the translocation between *S. cerevisiae* chromosomes VIII and XVI, found frequently in wine strains, was generated by an ectopic recombination between genes *ECM34*, a gene of unknown function, and *SSU1*, a gene encoding a sulfite pump, and resulted in a chimerical gene that confers greater resistance to sulfite, a preservative used during winemaking (**Figure 2A**). This recombination resulted in a new *SSU1* promoter that contained four repeats of a 76-bp sequence with putative binding sites for the transcription activator Fzf1p (**Figure 2B**). This translocation produced an enhanced expression for *SSU1*. These authors reported a perfect association between the sulfite resistance and the number of 76-bp repeated regions in the *SSU1* promoter (**Figure 2C**). In a recent QTL analysis study (Zimmer et al., 2014), another translocation between chromosomes XV and XVI has been related with a higher *SSU1* expression. This translocation is due to an ectopic recombination between the promoter regions of the genes *ADH1* and *SSU1*, and also produces an increase in the expression of *SSU1* during the first hours of alcoholic fermentation.

Chromosomal rearrangements are also involved in the postzygotic reproductive isolation between *Saccharomyces* species (Ryu et al., 1998). Although translocations may contribute to isolation (Delneri et al., 2003), they do not account by themselves for the isolation levels observed among *Saccharomyces* species (Fischer et al., 2000; Liti et al., 2006).

### Ploidy Changes

Aneuploidy, i.e., change in chromosome copy numbers, is originated by chromosomal non-disjunction during meiosis or mitosis, and generate a disproportion of gene products and the disruption of their interactions. Although it is one of the causes of their low sporulation levels, aneuploidy is, in general, tolerated by industrial yeasts and has been seen as an advantageous trait in yeasts because a higher number of gene copies may allow them to adapt to changing environments (Bakalinsky and Snow, 1990; Guijo et al., 1997).

Aneuploidies were detected originally by classical genetic analyses. Although most laboratory *Saccharomyces* strains appeared as diploid, higher aneuploidy levels have been described for certain industrial strains (Bakalinsky and Snow, 1990; Martínez et al., 1995; Guijo et al., 1997; Gallone et al., 2016). However, the development of array karyotyping (aCGH) and genome sequencing easily allowed the detection of whole chromosome aneuploidies in yeasts with contrasting results for wine strains. In this way, Infante et al. (2003) showed that *flor* yeasts were aneuploid for a few different chromosomes. However, in a similar study, Dunn et al. (2005) observed no aneuploidies in several commercial wine strains, including two of those previously described as aneuploid (Bakalinsky and Snow, 1990). Flow cytometry and microsatellite analyses of commercial wine yeasts have shown that most are diploid or almost diploid (Ayoub et al., 2006; Bradbury et al., 2006; Legras et al., 2007). This new evidence suggests that aneuploidy in wine strains is much less frequent than in other industrial strains such as brewing yeasts (Gallone et al., 2016).

The sequencing of the first *S. cerevisiae* complete genome revealed the presence of 376 duplicated genes in 55 large regions, which led Wolfe and Shields (1997) to postulate an ancient whole genome duplication event occurred in an ancestor of *S. cerevisiae* after its divergence from *K. lactis*, about 100–200 million years ago.

Genome duplication, or polyploidization, in yeasts can theoretically occur by several mechanisms (Morales and Dujon, 2012), classified as autopolyploidization when the result is a polyploid yeast, with four allelic copies of each chromosome from one single species, or as allopolyploidization (also called amphidiploidization) when the resulting polyploidy yeast contains several copies of chromosomes from two different species. Autopolyploidization can be generated by (i) non-disjunction during one of the meiotic divisions generates diploid spores, which can subsequently conjugate with other diploid or haploid spores to form tetra- or triploid cells; (ii) a non-disjunction during mitosis in unicellular organisms also produces tetraploid cells; (iii) a rare-mating event between two diploid cells or a diploid and a haploid cells from the same species, these diploid cells become mating-competent by a gene conversion at the MAT locus. Allotetraploidization can be generated by (i) interspecific hybridization by spore-to-spore conjugation, and subsequent genome duplication by non-disjunction either during mitosis or during meiosis; (ii) interspecific rare-mating between diploid cells or between diploid and haploid cells from different species.

The analysis of complete genomes sequences from species of the *Saccharomyces* complex confirmed that the whole genome duplication event encompassed the entire genome and was produced by allotetraploidization due to an ancient hybridization event (Marcet-Houben and Gabaldón, 2015).

The most important consequences of the whole genome duplication event were the sudden acquisition of extra copies of each gene, with slight differences due to the chimeric origin of the duplicated genome, and the provision of new gene functions that have profoundly impacted the evolution of the *Saccharomyces* lineage, particularly the adaptation of these species to highly efficient fermentation performance under anaerobic conditions and the development of efficient glucose-sensing and glucose-repression pathways (Piškur and Langkjær, 2004; Wolfe, 2004). The allotetraploidization event provided the basis for the evolution of new gene functions involved in the improvement of the fermentative performance and fast growth of the ancestors of *Saccharomyces* yeasts, which allow to their descendant industrial yeasts to become, under the selective pressures unconsciously imposed to improve controlled fermentation processes, today’s highly efficient mono- and oligosaccharide fermenters (Piškur et al., 2006).

### Interspecific Hybridization

In wine *Saccharomyces*, another remarkable mechanism of adaptation to fermentation environments is interspecific hybridization. Reproductive isolation among *Saccharomyces* species is mainly postzygotic, therefore, interspecific spore-to-spore or rare-mating crosses are possible. Although these interspecific hybrids are sterile, they are viable and can reproduce asexually by budding (Naumov, 1996; Sipiczki, 2008).

A well known example of interspecific hybrids are the lager yeasts *S. pastorianus* (syn. *S. carlsbergensis*) (Kodama et al., 2005), which are partial allotetraploid hybrids between *S. cerevisiae* and *S. eubayanus* (Libkind et al., 2011).

Natural hybrids also appear in wine fermentation, *S. uvarum* × *S. cerevisiae* hybrids have been isolated in wines from Italy (Masneuf et al., 1998); Alsace, France (Demuyter et al., 2004; Le Jeune et al., 2007) and Tokaj, Hungary (Antunovics et al., 2005). Other type of hybrids between *S. cerevisiae* and *S. kudriavzevii* are also present in wine fermentations of European regions with Continental and Oceanic climates (González et al., 2006; Lopandic et al., 2007; Erny et al., 2012; Peris et al., 2012a). González et al. (2006) also found a *S. bayanus* × *S. cerevisiae* × *S. kudriavzevii* hybrid isolated from Swiss wine.

By combining the phylogenetic analysis of gene sequences with all the information available on the genetic and genomic characterization of *S. cerevisiae* × *S. kudriavzevii* hybrids, seven potential hybridization events have been predicted as the origin of *S. kudriavzevii* wine hybrids (Peris et al., 2012b). These hybrids appear to have generated by rare-mating crosses between a diploid cell of wine *S. cerevisiae* strains and a haploid spore or cell of European *S. kudriavzevii* strains, because most hybrids contain triploid chimerical genomes (Erny et al., 2012; Peris et al., 2012c).

All *S. cerevisiae × S. kudriavzevii* natural hybrids analyzed so far predominantly maintained a *S. kudriavzevii* mitochondrial genome. The only exception is the commercial wine strain AMH, which has lost 69% of the nuclear genes of *S. kudriavzevii* coding for proteins involved in mitochondrial functions. Contrastingly, artificial hybrids obtained under non-selective pressures, inherited their mitochondrial genome from either one or the other parental species randomly (Solieri et al., 2008; Pérez-Través et al., 2014a). This discrepancy has been associated in other hybrids to adaptation to low temperatures (Rainieri et al., 2008), the influence of respiration levels (Solieri et al., 2008; Albertin et al., 2013) or to nuclear-mitochondrial incompatibilities (Lee et al., 2008).

Interestingly, some of these *S. cerevisiae × S. kudriavzevii* hybrids showed introgressions between both parental mtDNAs due to recombination in the mitochondrial *COX2* gene (Peris et al., 2012a), gene that has been used to determine mitochondrial inheritance in hybrids (González et al., 2006). Similar introgressions were also found in other hybrids (Pérez-Través et al., 2014b; Peris et al., 2014), and a recent study (Peris et al., 2017) demonstrated that these introgressions are very common among *Saccharomyces* species, which suggests extensive ancestral hybridization events during their evolutionary history.

**FIGURE 3:**
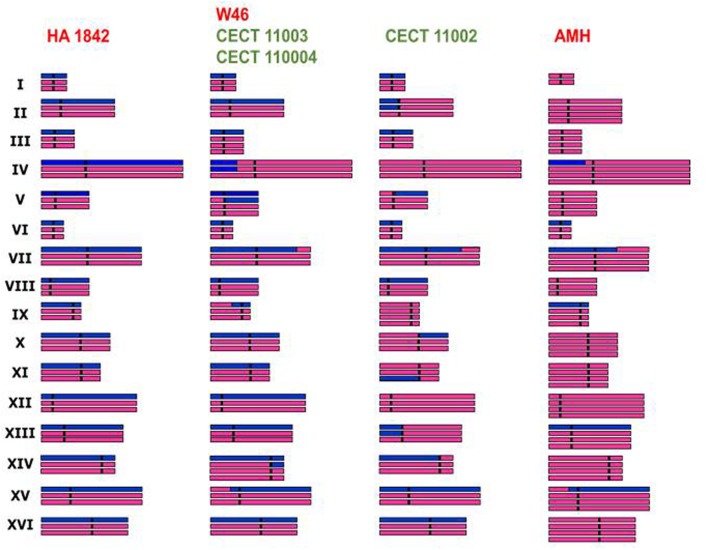
**Genome composition of natural *S. cerevisiae* × *S. kudriavzevii* hybrid representatives deduced from aCGH analysis, ploidy estimates and absence/presence of parental genes by RFLP analysis (Peris et al., 2012c).** Purple and blue bars are used to represent the *S. cerevisiae* and *S. kudriavzevii* genome fractions, respectively.

Genome sequencing and comparative genome hybridization demonstrated that *S. cerevisiae* × *S. kudriavzevii* hybrid strains contain aneuploidy differences and chimerical chromosomes that result from the recombination between “homeologous” chromosomes of different parental origin (Belloch et al., 2009; Borneman et al., 2012; Peris et al., 2012c) (**Figure 3**), promoting the loss of variable segments of the parental subgenomes. The evolution of hybrid genomes under stressful environmental conditions could make hybrid genomes to preserve chromosome rearrangements of selective value (Dunn et al., 2013). Therefore, interactions between both parental genomes, as well as between nuclear and mitochondrial genomes, together with the harsh environmental conditions present during fermentation, determine the final composition of hybrid genomes, which in the case of *S. cerevisiae* × *S. kudriavzevii* hybrids is characterized by the preservation of the *S. cerevisiae* subgenome and a progressive reduction of the *S. kudriavzevii* fraction (Peris et al., 2012c).

The enological characterization of natural hybrid strains *S. cerevisiae* × *S. uvarum* and *S. cerevisiae* × *S. kudriavzevii* has demonstrated that hybrids are well-adapted to ferment at low and intermediate temperatures, produce moderate or higher glycerol levels, and less acetic acid and more aromas (higher alcohols and esters) compared to *S. cerevisiae* and *S. kudriavzevii* reference strains (González et al., 2007; Gamero et al., 2013). The advantages of these hybrids can be correlated with their genome composition (Combina et al., 2012; Gamero et al., 2014; Pérez-Torrado et al., 2015).

### Horizontal Gene Transfer and Introgression

The comparative analysis of yeast genomes has shown the occurrence of genes present in a single yeast species or lineage for which the closest homologs are in bacteria (Hall et al., 2005). These genes, most of which encode metabolic enzymes, are rare in yeast genomes (<1%), but actually appear. By way of example, Gojkovic et al. (2004) demonstrated that a horizontal gene transfer (HGT) of a dihydroorotate dehydrogenase, from *Lactococcus lactis* to an ancestor of yeasts *Lachancea* and *Saccharomyces*, conferred them their capability to grow under anaerobic conditions.

Another example is the reacquisition of the biotin biosynthesis pathway in *Saccharomyces* yeasts. This pathway was lost in an ancestor of *S. cerevisiae*, but was later rebuilt by HGT from bacteria and subsequent gene neofunctionalization after duplication (Hall and Dietrich, 2007).

The genome of *S. cerevisiae* wine yeast EC1118 (Novo et al., 2009) showed the presence of three chromosomal segments acquired through independent HGT events from other yeast species. The donors of two of these regions were later identified. Region B, which comes from *Zygosaccharomyces bailii*, was inserted by means of a circular DNA (Galeote et al., 2011). A recent study (Marsit et al., 2015) has demonstrated that Region C, the largest one, derives from a recent transfer from *Torulaspora microellipsoides*. Marsit et al. (2015) demonstrated that the presence of *FOT* genes in this Region C, which facilitate the transport of the oligopeptides present in grape must, results in improved fermentation efficiency. Borneman et al. (2011) also observed a horizontally acquired cluster of five conserved ORFs that was present in most of the wine strains, encoding two potential transcription factors (one zinc-cluster, one C_6_ type), a cell surface flocullin, a nicotinic acid permease and a 5-oxo-L-prolinase.

For eukaryote-to-eukaryote HGT, unstable interspecific hybridization seems the most probable mechanism (Marinoni et al., 1999), although the unidirectional transfer of DNA from one nucleus to another in a newly formed hybrid prior to karyogamy has also been suggested (Morales and Dujon, 2012).

Such unstable interspecific hybridization can also explain the different events of introgression observed among *Saccharomyces* species (Liti et al., 2006; Dunn et al., 2012; Almeida et al., 2014). Some of these introgressed regions contain genes of adaptive value. Almeida et al. (2014) found in *S. uvarum* strains introgressed genome regions from *S. eubayanus*. These introgressed regions contain genes of the nitrogen metabolism, that might be advantageous in wine fermentation, in which nitrogen contents are limiting. Several *S. uvarum* strains isolated from New Zealand wines also contain introgressed regions from *S. eubayanus*. One of these regions comprises gene *FZF1*, encoding a transcription factor involved in the regulation of *SSU1*, the sulfite efflux pump gene. The presence of the *S. eubayanus FZF1* confers a higher tolerance to sulfite to these *S. uvarum* strains (Zhang et al., 2015). Recently, introgressions of the *SSU1* and *FZF1* genes from *S. paradoxus* to a wild Mediterranean population of *S. cerevisiae* have also been described (Almeida et al., 2017), which supports the adaptive value of introgressions.

## Methods to Detect Genetic Polymorphism

Traditionally, yeasts have been identified and classified by morphological and physiological traits (Kurtzman et al., 2011). These methods are laborious and time-consuming, and these characteristics have been influenced by culture conditions and can provide uncertain results (Yamamoto et al., 1991). Simplified biochemical methods have also been developed based on fermentation and assimilation characteristics (Rohm, 1990). Other methods have been based on the analysis of total proteins in the cell (Van Vuuren and Meer, 1987; Vancanneyt et al., 1991), isoenzymic patterns (Duarte et al., 1999), fatty acid analysis using gas chromatography (Cottrell et al., 1986; Tredoux et al., 1987; Moreira da Silva et al., 1994) or, more recently, the application of matrix-assisted laser desorption/ionization time of flight mass spectrometry (MALDI-TOF MS) for yeast differentiation (Blättel et al., 2013; Agustini et al., 2014), especially in the domain of medical sciences for the identification of pathogenic microorganisms (Stevenson et al., 2010; van Veen et al., 2010). However, DNA-based methods are currently the most widely used techniques for yeast differentiation. These techniques have the advantage of being independent of gene expression (Las Heras-Vazquez et al., 2003). Many molecular techniques have been developed to identify and characterize yeasts, such as DNA–DNA hybridization, electrophoretic karyotyping, restriction fragment length polymorphism (RFLP) and PCR-based methods. However, the irruption of next-generation sequencing (NGS) is revolutionizing the way for detecting genetic polymorphisms between organisms. NGS, also known as high-throughput sequencing, is the catch-all term used to describe a number of different modern sequencing technologies, which allow us to sequence DNA and RNA much more quickly and cheaply than Sanger sequencing.

Most studies into wine microbial ecology have invariably been conducted after culturing different microorganisms in distinct media. Nowadays, we witness a new era of microbiology due to the development of molecular biology techniques that allow us to identify and enumerate microorganisms using culture-independent methods. Avoiding the selective cultivation and isolation of microorganisms from natural samples is justified considering the biases related to traditional culture-dependent methods (Rantsiou et al., 2005). Presence of viable, but non-culturable, microorganisms has been described in wine samples (Millet and Lonvaud-Funel, 2000; Divol and Lonvaud-Funel, 2005). These microorganisms are unable to grow in plates, which may justify the differences reported by various authors between isolated and naturally occurring species in wine samples (Cocolin and Mills, 2003; Hierro et al., 2006b).

In this section, we discuss the most recent techniques for detecting genetic polymorphisms in wine yeasts. In wine microbial diversity studies, these techniques have been used mainly for *Saccharomyces* strains and have been used much less for non-*Saccharomyces* discrimination. Depending on the degree of polymorphism provided by the various molecular markers, they are more suitable for interspecific or for intraspecific discrimination. Therefore, we divided the molecular techniques into two main groups: those that can discriminate up to the species level and those that can discriminate up to the strain level.

### Methods for Monitoring Yeast Species Diversity during Winemaking

One of the most successful methods for yeast identification thanks to its rapidity and simplicity consists in the PCR amplification of ribosomal genes and the later restriction of the amplified fragment (PCR-RFLP). This technique is characterized by its easy execution and reproducibility. Guillamón et al. (1998) firstly adapted this technique to identify wine yeasts isolated from grape and wine fermentation processes. Later the restriction patterns of 191 yeast species were provided for the easy and reproducible identification of yeast isolated from food and fermentation processes (Esteve-Zarzoso et al., 1999; Fernández-Espinar et al., 2000; de Llanos-Frutos et al., 2004). To date, this method has been applied by numerous authors to study yeast biodiversity in grapes and wines (Torija et al., 2001; Beltrán et al., 2002; Hierro et al., 2006b; Ocón et al., 2010; Tello et al., 2012; Bezerra-Bussoli et al., 2013; Díaz et al., 2013).

Other independent-culture and PCR-based methods have also been applied for studying yeast species diversity during winemaking processes. This is the case of DGGE and real-time quantitative PCR. DGGE is a semi-quantitative technique based on the sequence-specific separation of PCR-derived rRNA gene amplicons in polyacrylamide gels that contain a linearly increasing concentration of denaturant (urea and formamide), as described by Muyzer (1999). Several authors have shown that DGGE is a well-suited technique for studying yeast population dynamics during wine fermentation (Cocolin et al., 2000; Prakitchaiwattana et al., 2004; Di Maro et al., 2007; Renouf et al., 2007; Stringini et al., 2009; Pérez-Martín et al., 2014), as well as the impact of different viticultural and enological techniques in this diversity (Nisiotou and Nychas, 2007; Andorrà et al., 2008; Milanović et al., 2013). A related technique is temperature gradient gel electrophoresis (TGGE), based on a linear temperature gradient for separating DNA molecules. TGGE has also been applied to the characterization of wine yeasts (Hernán-Gómez et al., 2000; Manzano et al., 2005). However, these methods have their drawbacks: they cannot discriminate between live and dead microorganisms and minor microorganisms go undetected when they co-exist with overwhelming species populations (Andorrà et al., 2008). A modification to the DGGE method has been recently proposed by Takahashi et al. (2014) to identify low-abundant eukaryotic microorganisms. These authors modified the co-amplification at lower denaturation temperature PCR (COLD-PCR) method used to detect minor SNPs that co-exist with an overwhelming majority of wild-type (WT) sequences, as proposed by Li et al. (2008). By combining this modified COLD-PCR with DGGE (mCOLD-PCR-DGGE), these authors detected low-abundant microorganisms more efficiently, even when a specific microorganism represented an overwhelming majority of the sample. *Schizosaccharomyces pombe* was detected in a model sample that co-existed with 10000 times as many *S. cerevisiae*. When mCOLD-PCR-DGGE was applied in a microbiota analysis of a fermenting white wine, *Candida* sp. and *Cladosporium* sp. were detected that were not detected by conventional PCR-DGGE.

Real-time PCR offers numerous advantages compared to other identification techniques. It is worth stressing its high specificity and sensitivity, its ability to quantify and the fact that no analysis after PCR is necessary (electrophoresis). qPCR can even be multiplexed to detect a number of organisms in one assay (Selma et al., 2009). This technique has been developed to detect and quantify total yeasts (Hierro et al., 2006a), *Brettanomyces* (Phister and Mills, 2003; Delaherche et al., 2004; Tofalo et al., 2012; Willenburg and Divol, 2012; Vendrame et al., 2014), *Hanseniaspora* (Hierro et al., 2007; Phister et al., 2007), *Saccharomyces* (Martorell et al., 2005b; Hierro et al., 2007; Salinas et al., 2009), and *Zygosaccharomyces* (Rawsthorne and Phister, 2006) in wine and other fermentation processes. The main disadvantage other than cost and personnel training lies in the method’s inability to differentiate viable and non-viable microbes (Ivey and Phister, 2011). Several possible solutions have been indicated to overcome the detection of non-viable microorganisms; e.g., using RNA as a target for PCR amplification (Bleve et al., 2003; Hierro et al., 2006a) because, in theory, RNA is much more unstable than DNA, and is considered an indicator of viability; or using a fluorescent photoaffinity label which covalently couples to nucleic acids upon exposure to light, such as EMA and PMA (Andorrà et al., 2010). These dyes can only enter cells with compromised cell walls and cell membranes, and thus remove DNA from dead cells and then quantify only live microorganisms. However, this and other techniques are being replaced with the power of NGS techniques.

The determination and comparison of the nucleotide sequences of different yeast genome regions is a very useful tool for identifying and inferring phylogenetic relationships between different yeast species. The two most commonly used regions are those that correspond to domains D1 and D2 located at the 5′ end of gene 26S (Kurtzman and Robnett, 1998) and gene 18S (James et al., 1997). The availability of these sequences in databases, especially for the D1/D2 region of gene 26S, makes this technique very useful for assigning an unknown yeast to a specific species when the percentage of homology of its sequences is over or similar to 99% (Kurtzman and Robnett, 1998). However, some authors have advocated the use of multilocus sequence analyses (MLSA) for yeast identification (Kurtzman and Robnett, 2003; Tavanti et al., 2005). These sequences were obtained by the Sanger method. However, since 2005, the NGS methods have emerged and replaced previous techniques because the sequence data generated from a single experiment are immensely more numerous. NGS tools enable the sensitive profiling of microbial communities on an unprecedented scale by the massively parallel sequencing of short (100- to 600-bp) DNA fragments amplified by PCR. The large number of sequences delivered by a single NGS run (10^4^ to 10^8^ reads) allows a more sensitive description of diverse microbial communities and greater multiplexing, which means a greater per-run sequencing capacity (Bokulich et al., 2014). This technology has been recently applied to study microbial diversity in grapes and wine by metagenomics approaches. Metagenomic surveillances have revealed higher diversity than other community fingerprinting methods and culture-based methods (David et al., 2014; Taylor et al., 2014). In fact, Taylor et al. (2014) suggested that culture-based methods might miss up to approximately 95% of the community in some samples. Consequently, these methods are increasingly becoming the preferred tool to evaluate grape microbial community structures. Bokulich et al. (2014) comprehensively examined the communities of both bacteria and fungi in crushed Chardonnay and Cabernet Sauvignon fruit in California by Illumina amplicon sequencing approaches and showed that microbiomes not only differed by region, but were also conditioned by climate, year, and cultivar. Similarly, Taylor et al. (2014) demonstrated regional distinction in fungal communities in vineyards across New Zealand. The diversity of yeasts associated with grapes and present in grape must have been shown to resemble that present on leaves (Bokulich et al., 2014; Pinto et al., 2014), and community composition to be influenced by chemical treatments, agronomic practices, and climatic conditions (Setati et al., 2012, 2015; Bokulich and Mills, 2013; David et al., 2014; Pinto et al., 2014). Setati et al. (2015) compared the mycobiome associated with South African (SA) Cabernet Sauvignon grapes in three neighboring vineyards that employed different agronomic approaches by a sequence-based metagenomic approach. The data revealed approximately 10-fold more fungal diversity than what is typically retrieved from culture-based studies.

Similar studies are reported in the literature about monitoring yeast biodiversity in must and during alcoholic fermentation. Pinto et al. (2015) characterized the wine microbiome from six Portuguese wine appellations. The wine fermentation process revealed a stronger impact on yeast populations compared with bacterial communities, and fermentation evolution clearly caused loss of environmental microorganisms. Interestingly, a biogeographical correlation for both yeast and bacterial communities was identified between wine appellations, which suggests that each wine region contains specific embedded microbial communities that might contribute to the uniqueness of regional wines. In a similar metagenomics study conducted during the spontaneous fermentation of “Vino Santo Trentino,” Stefanini et al. (2016a) also suggested the existence of a highly winery-specific “microbial-terroir” during fermentation that could contribute significantly to the final product rather than a regional “terroir.” This indication was extended to human-related environments through the observation already made in wild environments; namely microbial populations are influenced more by microevolution in their ecological niche than by their geographical location (Morrison-Whittle and Goddard, 2015).

It is noteworthy that two recent studies compared pyrosequencing technology with some of the above-mentioned methods for studying yeast diversity during winemaking, PCR-RFLP, quantitative PCR and DGGE (David et al., 2014; Wang et al., 2015). David et al. (2014) evidenced the power of NGS technology and the drawback of the former techniques for monitoring microbial diversity. DGGE proved unsuitable for the quantification of biodiversity and its use for species detection was limited by the initial abundance of each species. The isolates identified by PCR-RFLP were not fully representative of the true population. For population dynamics, high-throughput sequencing technology yielded results that differed in some respects from those obtained by other approaches. Wang et al. (2015) reached similar conclusions; massive sequencing was more appropriate for understanding the fungal community in grape must after crushing than the other techniques used in this study. They also concluded that the “*terroir*” characteristics of the fungus population related more to vineyard location than to grape variety.

### Methods for Fingerprinting Yeast Strain Diversity during Winemaking

Fingerprinting generally examines the whole genome of an organism by often creating a banding pattern by digesting or amplifying genome regions that can be compared between organisms (Ivey and Phister, 2011). Fingerprinting methods are characterized because they present a sufficient degree of genetic polymorphism to differentiate between strains of the same yeast species. As not all the strains of a species present the same industrial traits, availability of techniques that can discriminate at the inter- and intraspecific levels is important. As mentioned for species-differentiation techniques, new genotyping by sequencing (GBS) methods is seen as future strain genotyping. However, many studies that have compared strains still rely on some type of fingerprinting as they provide rapid and less expensive alternatives (Ivey and Phister, 2011). Although many molecular methods have been developed for yeast strain typing, most have been exclusively applied to *Saccharomyces* strains, although the literature offers some non *Saccharomyces* typing examples.

#### Restriction Analysis of Mitochondrial DNA

The mtDNA of *S. cerevisiae* is a small molecule of between 65 and 80 kb, whose degree of variability can be shown by restriction. The high degree of polymorphism revealed by this technique among *S. cerevisiae* strains means that it is one of the most commonly applied techniques to characterize reference and commercial wine yeast strains (Querol et al., 1992; Guillamón et al., 1996; Fernández-Espinar et al., 2001; Esteve-Zarzoso et al., 2004; Schuller et al., 2004). This technique has been much more limited in typing strains that belong to other species, but some applications can be found in the literature to differentiate *Candida* spp. (Romano et al., 1996; Sabate et al., 2002), *Zygosaccharomyces* (Guillamón et al., 1997; Esteve-Zarzoso et al., 2003), *D. bruxellensis* and *P. guilliermondii* (Martorell et al., 2006) and *Kluyveromyces* (Belloch et al., 1997) strains. So although RFLP mtDNA analyses have shown narrow variability and limited usefulness for some species, it is an efficient technique for differentiating at the strain level in many other yeast species. At present, the *S. cerevisiae* mtDNA variability can also be analyzed by NGS methods (Wolters et al., 2015).

#### PCR Technique-Based Methods

The PCR technique has made available rapid methods to discriminate wine yeast strains. These methods detect the genetic polymorphism by amplifying different yeast genome regions. Amplified fragments are further separated in an agarose gel to obtain an exclusive banding pattern for each strain.

The Randomly Amplified Polymorphic DNA (RAPD-PCR) fingerprint amplifies genomic DNA with a single primer of arbitrary sequence, 9 or 10 bases in length, which hybridize with sufficient affinity to chromosomal DNA sequences at low annealing temperatures. The result is a pattern of amplified products of different molecular weights that can be characteristic of either the species or the different strains or isolates within the same species (Bruns et al., 1991; Paffetti et al., 1995). This technique has been successfully applied to differentiate wine yeast strains belonging to different species (Quesada and Cenis, 1995; Baleiras Couto et al., 1996; Romano et al., 1996; Tornai-Lehoczki and Dlauchy, 2000; Čadež et al., 2002; Martínez et al., 2004; Gallego et al., 2005; Martorell et al., 2006; Urso et al., 2008; Pfliegler et al., 2014).

Although yeast genomes are not very rich in repetitive sequences compared with higher eukaryotes, the recent sequencing of entire yeast genomes has revealed the presence of different repetitive regions. The use of primers based on conserved sequences of these repeated regions has proven most useful for strain differentiation by PCR. Microsatellites are short (usually less than 10-bp) sequence repeats that have been shown to exhibit a substantial level of polymorphism in a number of eukaryotic genomes (Hennequin et al., 2001). The variability found in these regions can be shown by PCR amplification using specific oligonucleotides, such as (GTG)_5_, (GAG)_5,_ (GACA)_4_ or M13. The ability of these oligonucleotides to reveal polymorphisms among *S. cerevisiae* strains has been demonstrated by Lieckfeldt et al. (1993) by hybridization techniques. These same authors were the first to use these sequences as primers in a PCR reaction, and proved the usefulness of this technique for characterization at the strain level. It has later been used by other authors for typing *Saccharomyces* (Baleiras Couto et al., 1996; Pérez et al., 2001; Howell et al., 2004; Schuller et al., 2004; Masneuf-Pomarède et al., 2007), non-*Saccharomyces* (Capece et al., 2003), *Brettanomyces* (Miot-Sertier and Lonvaud-Funel, 2007), *Hanseniaspora* (Caruso et al., 2002), and *Zygosaccharomyces* (Martorell et al., 2005a) strains.

Delta (δ) sequences are elements which measure the 0.3 kb that flank retrotransposons Ty1 (Cameron et al., 1979). Around 100 δ copies are present in the yeast genome of *S. cerevisiae* as part of retrotransposons Ty1 or as isolated elements. The number and localization of these elements demonstrate certain intraspecific variability, which Ness et al. (1993) took advantage of to develop specific primers (δ_1_ and δ_2_) that are useful to differentiate strains of *S. cerevisiae.* Later Legras and Karst (2003) optimized the technique by designing two new primers (δ_12_ and δ_21_) located very near δ_1_ and δ_2_. The use of either δ_12_ and δ_21_ or δ_12_ with δ_2_ revealed a greater polymorphism as reflected by the appearance of more bands. Consequently, new primers were able to differentiate more strains, and 53 commercial strains were unequivocally differentiated (Legras and Karst, 2003). Schuller et al. (2004) confirmed this later by showing that the δ_2_ and δ_12_ combination could identify twice as many strains as the set of primers designed by Ness et al. (1993).

Approximately 5% of *S. cerevisiae* genes possess introns. These introns are spliced from pre-mRNA to form functional mature mRNAs during a process that requires the spliceosome, a large ribonucleoprotein complex. A conserved sequence is present in the intron structure for spliceosome recognition. De Barros Lopes et al. (1996) designed primers based on these conserved sequences, known as intron splice sites (ISS). The use of these primers has enabled the differentiation of a large number of commercial wine strains. ISS primers can also be used with non-*Saccharomyces* strains because ISS are conserved in all the yeasts that have been studied to date. Hierro et al. (2004) used these primers to identify wine strains that belong to 15 different species. This technique has also been applied to genotype *B. bruxellensis* strains (Oelofse et al., 2009; Vigentini et al., 2012).

Amplified Fragment Length Polymorphism (AFLP) is a technique that involves the restriction of genomic DNA, followed by binding adapters to the obtained fragments and their selective PCR amplification. The adapter sequence and restriction sites are used as target primers for PCR amplification. Amplified fragments are separated in polyacrylamide gels and different genotypes display an exclusive banding pattern (Vos et al., 1995). AFLP is a useful technique for discriminating between wine yeasts at the strain level, as shown by de Barros Lopes et al. (1999) and other authors (Azumi and Goto-Yamamoto, 2001; Boekhout et al., 2001; Lopandic et al., 2007). However, its drawback is that it is very laborious, requires automatic sequencers, highly sophisticated for the wine industry, and the results obtained are also difficult to interpret. To overcome this drawback, Esteve-Zarzoso et al. (2010) developed a simplified AFLP method that allowed gel electrophoresis analyses and considerably reduced equipment requirements. Another remarkable improvement was to use non-labeled primers that reduces analysis costs. This simplified method was applied to the reference strains and colonies isolated from the spontaneous fermentation of species *H. uvarum*, *H. vinae*, *C. zemplinina*, and *S. cerevisiae*. Recently, this technique has been used also to characterize genetic variability within the *H. uvarum* species (Albertin et al., 2015).

## Author Contributions

Both authors have contributed to the writing, revision, and final edition of this review.

## Conflict of Interest Statement

The authors declare that the research was conducted in the absence of any commercial or financial relationships that could be construed as a potential conflict of interest.
